# Supplementary material for: 3D organoid-derived human glomeruli for personalised podocyte disease modelling and drug screening
Source: Nat Commun. 2018 Dec 4;9:5167. doi: 10.1038/s41467-018-07594-z (PMC6279764; doi:10.1038/s41467-018-07594-z)
Supplement: Supplementary file 1 — Supplementary Information [file 41467_2018_7594_MOESM1_ESM.pdf]

Supplementary data

3D organoid-derived human glomeruli for personalised podocyte disease modelling and drug screening.

Lorna J. Hale, Sara E. Howden, Belinda Phipson, Andrew Lonsdale, Pei X. Er, Irene Ghobrial, Salman Hosawi, Sean Wilson, Kynan Lawlor, Shahnaz Khan, Alicia Oshlack, Catherine Quinlan, Rachel Lennon, Melissa H. Little

Contents

Supplementary Tables 1-4

Supplementary Figures 1-6

Supplementary Video 1

Supplementary references

Supplementary Table 1.

Symbol	Gene name	logFC	*P* Value
CDKN1A	Cyclin dependent kinase inhibitor 1A	4.085346	4.60E-14
PDZK1IP1	PDZK1 interacting protein 1	5.862841	6.45E-13
TRIM22	Tripartite motif containing 22	3.559167	1.02E-10
TNFRSF10B	TNF receptor superfamily member 10b	2.509024	1.46E-10
C3	Complement C3	8.140257	1.48E-10
CLDN1	Claudin 1	3.55235	2.66E-10
PHLDA1	Pleckstrin homology like domain family A member 1	3.311057	8.52E-10
PBXIP1	PBX homeobox interacting protein 1	2.216353	2.12E-09
EDA2R	Ectodysplasin A2 receptor	4.138096	2.47E-09
TMEM159	Transmembrane protein 159	3.193489	1.08E-08
FUCA1	Fucosidase, alpha-L- 1, tissue	2.381889	1.22E-08
NRP2	Neuropilin 2	2.440631	3.27E-08
CSF1	Colony stimulating factor 1	2.518191	5.04E-08
SUSD6	Sushi domain containing 6	2.583393	5.78E-08
SOWAHB	Sosondowah ankyrin repeat domain family member B	3.912102	1.26E-07
GDF15	Growth differentiation factor 15	6.631471	1.47E-07
IL6	Interleukin 6	5.364979	1.83E-07
DRAM1	DNA damage regulated autophagy modulator 1	3.06241	2.11E-07
BTG2	BTG anti-proliferation factor 2	3.541625	2.32E-07
ATG16L2	Autophagy related 16 like 2	3.300185	2.37E-07
TPCN1	Two pore segment channel 1	2.927092	2.94E-07
TNFSF10	TNF superfamily member 10	5.100832	2.98E-07
SEL1L3	SEL1L family member 3	2.515535	3.13E-07
TRANK1	Tetratricopeptide repeat and ankyrin repeat containing 1	3.54394	3.61E-07
SULF2	Sulfatase 2	7.209275	6.50E-07
PLAU	Plasminogen activator, urokinase	3.675696	7.17E-07
MANBA	Mannosidase beta	2.464472	7.60E-07
CD82	CD82 molecule	4.16917	1.22E-06
JUP	Junction plakoglobin	2.868247	1.23E-06
CDKN1A	Cyclin dependent kinase inhibitor 1A	4.085346	4.60E-14

Footnote: The top 30 most differentially upregulated genes following induced differentiated at 37°C.

Supplementary Table 2.

UNIPROT	Description	Gene Symbol	OrgGlom (C4) ion intensity	OrgPT (C4) ion intensity	Max FC	Anova (*p*)	Division	Category
O00468	Agrin	AGRN	89.87	40.52	2.22	0.08692	Core Matrisome	ECM Glycoproteins
O00468	Agrin (fragment)	AGRN	5.93	5.21	1.14	0.93235	Core Matrisome	ECM Glycoproteins
P39060	Collagen, type XVIII, alpha 1, isoform CRA_d	COL18A1	215.94	232.26	0.93	0.74107	Core Matrisome	Collagens
P02452	Collagen, type I, alpha 1, isoform CRA_a	COL1A1	18.76	17.13	1.09	0.70141	Core Matrisome	Collagens
P02452	Collagen alpha-1(I) chain	COL1A1	182.72	265.91	0.69	0.25455	Core Matrisome	Collagens
P08123	Collagen alpha-2(I) chain	COL1A2	14.95	24.48	0.61	0.21043	Core Matrisome	Collagens
P02458	Collagen alpha-1(II) chain	COL2A1	20.05	36.08	0.56	0.08036	Core Matrisome	Collagens
P02462	Collagen alpha-1(IV) chain	COL4A1	185.21	3.36	55.19	0.00004	Core Matrisome	Collagens
P08572	Collagen, type IV, alpha 2, isoform CRA_a	COL4A2	152.12	73.31	2.07	0.04782	Core Matrisome	Collagens
Q01955	Collagen alpha-3(IV) chain	COL4A3	13.06	13.53	0.96	0.80129	Core Matrisome	Collagens
P29400	Collagen alpha-5(IV) chain	COL4A5	269.55	409.16	0.66	0.02797	Core Matrisome	Collagens
Q14031	Collagen alpha-6(IV) chain	COL4A6	117.94	107.53	1.10	0.80216	Core Matrisome	Collagens
P12109	Collagen alpha-1(VI) chain	COL6A1	6.06	7.36	0.82	0.22733	Core Matrisome	Collagens
P12110	Collagen alpha-2(VI) chain	COL6A2	15.08	3.90	3.86	0.07267	Core Matrisome	Collagens
P12111	Collagen alpha-3(VI) chain	COL6A3	25.68	79.46	0.32	0.13462	Core Matrisome	Collagens
P07339	Cathepsin D	CTSD	1.29	3.84	0.34	0.00250	Matrisome-Assoc.	ECM Regulators
Q86XX4	Extracellular matrix protein FRAS1	FRAS1	25.91	7.81	3.32	0.20262	Core Matrisome	ECM Glycoproteins
Q5SZK8	FRAS1-related extracellular matrix protein 2	FREM2	34.23	3.40	10.06	0.00503	Matrisome-Assoc.	ECM-affiliated Proteins
P43026	GDF5 (fragment)	GDF5	115.28	96.54	1.19	0.37924	Matrisome-Assoc.	Secreted Factors
P10915	Hyaluronan and proteoglycan link protein 1, isoform, CRA_a	HAPLN1	46.68	79.82	0.58	0.38245	Core Matrisome	Proteoglycans
P51610	Host cell factor 1	HCFC1	15.64	34.72	0.45	0.03141	Matrisome-Assoc.	Secreted Factors
Q8NDA	Hemicentin-2	HMCN2	32.36	8.71	3.72	0.08496	Core Matrisome	ECM Glycoproteins
P98160	Heparan sulfate proteoglycan 2 (Perlecan), isoform CRA_b	HSPG2	35.00	16.86	2.08	0.03036	Core Matrisome	Proteoglycans
Q92743	HTRA1 protein (fragment)	HTRA1	1.31	1.48	0.88	0.44501	Matrisome-Assoc.	ECM Regulators
Q16270	Insulin-like growth factor binding protein 7 (Fragment)	IGFBP7	52.27	2.49	21.02	0.00139	Core Matrisome	ECM Glycoproteins
P25391	Laminin subunit alpha-1	LAMA1	9.42	5.74	1.64	0.16871	Core Matrisome	ECM Glycoproteins
Q16787	Laminin subunit alpha-3	LAMA3	21.12	11.43	1.85	0.12774	Core Matrisome	ECM Glycoproteins
O15230	Laminin subunit alpha-5	LAMA5	115.69	4.46	25.93	0.00001	Core Matrisome	ECM Glycoproteins
P07942	Laminin subunit beta-1	LAMB1	30.1	0.61	49.03	0.01163	Core Matrisome	ECM Glycoproteins
P55268	Laminin, beta 2 (Laminin S), isoform CRA_a	LAMB2	74.18	2.09	35.51	0.00242	Core Matrisome	ECM Glycoproteins
P11047	Laminin subunit beta-4	LAMB4	4.42	7.45	0.59	0.39654	Core Matrisome	ECM Glycoproteins
A4D0S4	Laminin, gamma 1 (Formerly LAMB2), isoform CRA_a	LAMC1	143.38	2.91	49.30	0.00007	Core Matrisome	ECM Glycoproteins
Q08397	Lysyl oxidase homolog 1	LOXL1	14.18	13.37	1.06	0.65604	Matrisome-Assoc.	ECM Regulators
P55081	Microfibrillar-associated protein 1	MFAP1	14.31	20.20	0.71	0.15588	Core Matrisome	ECM Glycoproteins
Q08431	Milk fat globule-EGF factor 8 protein, isoform CRA_a	MFGE8	24.11	11.77	2.05	0.05612	Core Matrisome	ECM Glycoproteins
P14543	Nidogen 1	NID1	87.75	1.90	46.25	0.00002	Core Matrisome	ECM Glycoproteins
Q8IVN8	Somatomedin-B and thrombospondin type-1 domain containing protein	SBSPON	28.20	1.14	24.78	0.00017	Core Matrisome	ECM Glycoproteins
P50454	Serpin peptidase inhibitor, clade H (HSP47), member 1, (CBP1)	SERPINH1	249.18	26.06	9.56	0.00152	Matrisome-Assoc.	ECM Regulators
O94813	Slit homolog 2 protein	SLIT2	25.58	20.82	1.23	0.54663	Core Matrisome	ECM Glycoproteins
P21980	Protein-glutamine gamma-glutmyltransferase 2	TGM2	6.21	2.77	2.24	0.02147	Matrisome-Assoc.	ECM Regulators
O43548	Protein-glutamine gamma-glutmyltransferase 5	TGM5	8.41	4.54	1.85	0.50593	Matrisome-Assoc.	ECM Regulators
Q9GZM7	Tubulointerstitial nephritis antigen-like	TINAGL1	0.84	0.09	9.05	0.21190	Core Matrisome	ECM Glycoproteins

Footnote: Enriched extracellular matrix (C4 fraction) enriched proteins identified by mass spectrometry.

Supplementary Table 3.

UNIPROT	Description	Gene Symbol	OrgGlom (C4) ion intensity	OrgPT (C4) ion intensity	Max FC	Anova (*p*)	Division	Category
O14672	ADAM metallopeptidase domain 10, isoform CRA_b	ADAM10	13.51	16.47	0.82	0.19109	Matrisome-Assoc.	ECM Regulators
O00468	Agrin	AGRN	13.71	3.10	4.42	0.04029	Core Matrisome	ECM Glycoproteins
P04083	Annexin A1	ANXA1	21.07	51.37	4.13	0.00029	Matrisome-Assoc.	ECM-affiliated Proteins
P07355	Annexin	ANXA2	376.20	178.74	2.10	0.00007	Matrisome-Assoc.	ECM-affiliated Proteins
P09525	Annexin A4	ANXA4	106.96	154.44	0.69	0.00041	Matrisome-Assoc.	ECM-affiliated Proteins
P08758	Annexin A5	ANXA5	123.11	85.33	1.44	0.06088	Matrisome-Assoc.	ECM-affiliated Proteins
P12109	Collagen alpha-1(VI) chain	COL6A1	10.43	15.67	0.67	0.37976	Core Matrisome	Collagens
A8TX70	Collagen alpha-5(VI) chain	COL6A5	36.67	20.25	1.81	0.0088	Core Matrisome	Collagens
Q02388	Collagen alpha-1(VII) chain	COL7A1	10.18	12.07	0.84	0.52353	Core Matrisome	Collagens
P53634	Dipeptidyl peptidase 1	CTSC	66.91	49.81	1.34	0.80944	Matrisome-Assoc.	ECM Regulators
P53634	Dipeptidyl peptidase 1 (fragment)	CTSC	4.52	1.82	2.48	0.25719	Matrisome-Assoc.	ECM Regulators
P07339	Cathepsin D	CTSD	280.35	227.53	1.23	0.00218	Matrisome-Assoc.	ECM Regulators
Q9UBR2	Cathepsin Z	CTSZ	21.82	24.20	0.90	0.08967	Matrisome-Assoc.	ECM Regulators
P51610	Host ecll factor 1	HCFC1	36.52	51.98	0.70	0.17547	Matrisome-Assoc.	Secreted Factors
P98160	Basement membrane-specific heparan sulfate proteoglycan core protein	HSPG2	16.69	10.29	1.62	0.01327	Core Matrisome	Proteoglycans
Q16270	Insulin-like growth factor binding protein 7, isoform CRA_a (fragment)	IGFBP7	6.54	6.67	0.98	0.78312	Core Matrisome	ECM Glycoproteins
P19823	Inter-alpha-trypsin inhibitor heavy chain H2	ITIH2	22.99	25.44	0.90	0.58236	Matrisome-Assoc.	ECM Regulators
O15230	Laminin subunit alpha-5	LAMA5	2456.96	2831.03	0.87	0.20977	Core Matrisome	ECM Glycoproteins
P55268	Laminin, beta 2 (Laminin S), isoform CRA_a	LAMB2	10.49	15.37	0.68	0.13858	Core Matrisome	ECM Glycoproteins
P11047	Laminin, gamma 1 (Formerly LAMB2), isoform CRA_a	LAMC1	59.22	10.48	5.65	0.00060	Core Matrisome	ECM Glycoproteins
P49257	Lectin, mannose-binding 1, isoform CRA_b	LMAN1	0.45	44.13	0.01	0.02840	Matrisome-Assoc.	ECM-affiliated Proteins
P14543	Nidogen-1	NID1	43.75	3.64	12.02	0.00004	Core Matrisome	ECM Glycoproteins
Q14112	Nidogen-2	NID2	6.07	1.66	3.66	0.00264	Core Matrisome	ECM Glycoproteins
P13674	Procollagen-proline, 2-oxoglutarate 4-dioxygenase, alpha polypeptide I	P4HA1	39.42	60.43	0.65	0.01261	Matrisome-Assoc.	ECM Regulators
O15460	Prolyl 4-hydroxylase subunit alpha-2	P4HA2	5.65	3.95	1.43	0.25057	Matrisome-Assoc.	ECM Regulators
Q02809	Procollagen-lysine 1, 2-oxoglutarate 5-dioxygenase 1, isoform CRA_a	PLOD1	20.33	17.1	1.19	0.05547	Matrisome-Assoc.	ECM Regulators
O00469	Procollagen-lysine, 2-oxoglutarate 5-dioxygenase 2	PLOD2	96.00	76.96	1.25	0.05608	Matrisome-Assoc.	ECM Regulators
O60568	Procollagen-lysine, 2-oxoglutarate 5-dioxygenase 3	PLOD3	20.41	25.65	0.80	0.09117	Matrisome-Assoc.	ECM Regulators
O15031	Plexin B-2	PLXNB2	35.33	34.24	1.03	0.67752	Matrisome-Assoc.	ECM-affiliated Proteins
P35237	Serpin peptidase inhibitor, clade B, member 6, isoform CRA_a	SERPINB6	18.30	9.30	1.97	0.00529	Matrisome-Assoc.	ECM Regulators
P50453	Serpin peptidase inhibitor, clade B, member 9, isoform CRA_a	SERPINB9	99.45	62.04	1.60	0.64945	Matrisome-Assoc.	ECM Regulators

Footnote: Enriched cellular and vesicular proteins (C1 fraction) identified by mass spectrometry.

Supplementary Table 4.

Symbol	Gene name	Max FC	*P* Value	Association
KDR	Kinase insert domain receptor	23.93	1.94E-03	Podocyte / Endothelial
COL4A4	Collagen Type IV alpha 4 chain	15.89	1.72E-04	GBM
MMP2	Matrix metallopeptidase 2	8.61	1.65E-05	GBM / Mesangium
CXCL12	Chemokine (C-X-C motif) ligand 12	8.38	8.87E-03	Renal vasculature
COL4A3	Collagen Type IV alpha 3 chain	7.71	1.73E-03	GBM
ITGA2	Integrin, alpha 2	6.85	4.49E-03	GBM / matrices
TEK	TEK receptor tyrosine kinase (TIE2)	6.34	1.31E-04	Endothelial
FN1	Fibronectin 1	6.26	3.10E-03	Glomerular
ANGPTL2	Angiopoietin like 2	5.41	1.76E-03	Endothelial / vessels
IGFBP3	Insulin like growth factor binding protein 3	4.25	1.45E-05	Podocyte
SHISA3	Shisa homolog 3	4.19	5.16E-05	Nephron progenitors
MME	Membrane metallo-endopeptidase (CD10)	3.97	8.52E-05	Podocyte
IGFBP5	Insulin like growth factor binding protein	3.61	19.7E-05	Mesangium
EMCN	Endomucin	3.21	3.27E-05	Endothelial
UCHL1	Ubiquitin C-terminal hydrolase L1	2.74	5.13E-03	Podocyte / PEC
TAGLN	Transgelin 2	2.74	1.94E-04	Mesangium
CDH5	Cadherin 5	2.66	9.40E-02	Endothelial
GJA5	Gap junction protein alpha 5	2.57	1.78E-03	Endothelial / vessels
SMAD7	SMAD family member 7	2.38	4.26E-05	Glomerular
CX3CL1	C-X3-C motif chemokine ligand 1	2.32	3.46E-05	Glomerular
FGF2	Fibroblast growth factor 2	2.30	1.18E-02	Glomerular
GATA3	GATA binding protein 3	2.25	1.35E-02	Glomerular
DES	Desmin	2.19	8.66E-03	Mesangium
FOXD1	Forkhead box D1	2.01	8.68E-03	Mesangium
C1QTNF12	C1q and TNF related 12	2.00	1.49E-02	Podocyte

Footnote: Represents the most differentially expressed glomerular-specific genes [1] identified between MAFB-mTAGBFP sorted cells isolated at d7+10 and organoid glomeruli isolated at d7+19.

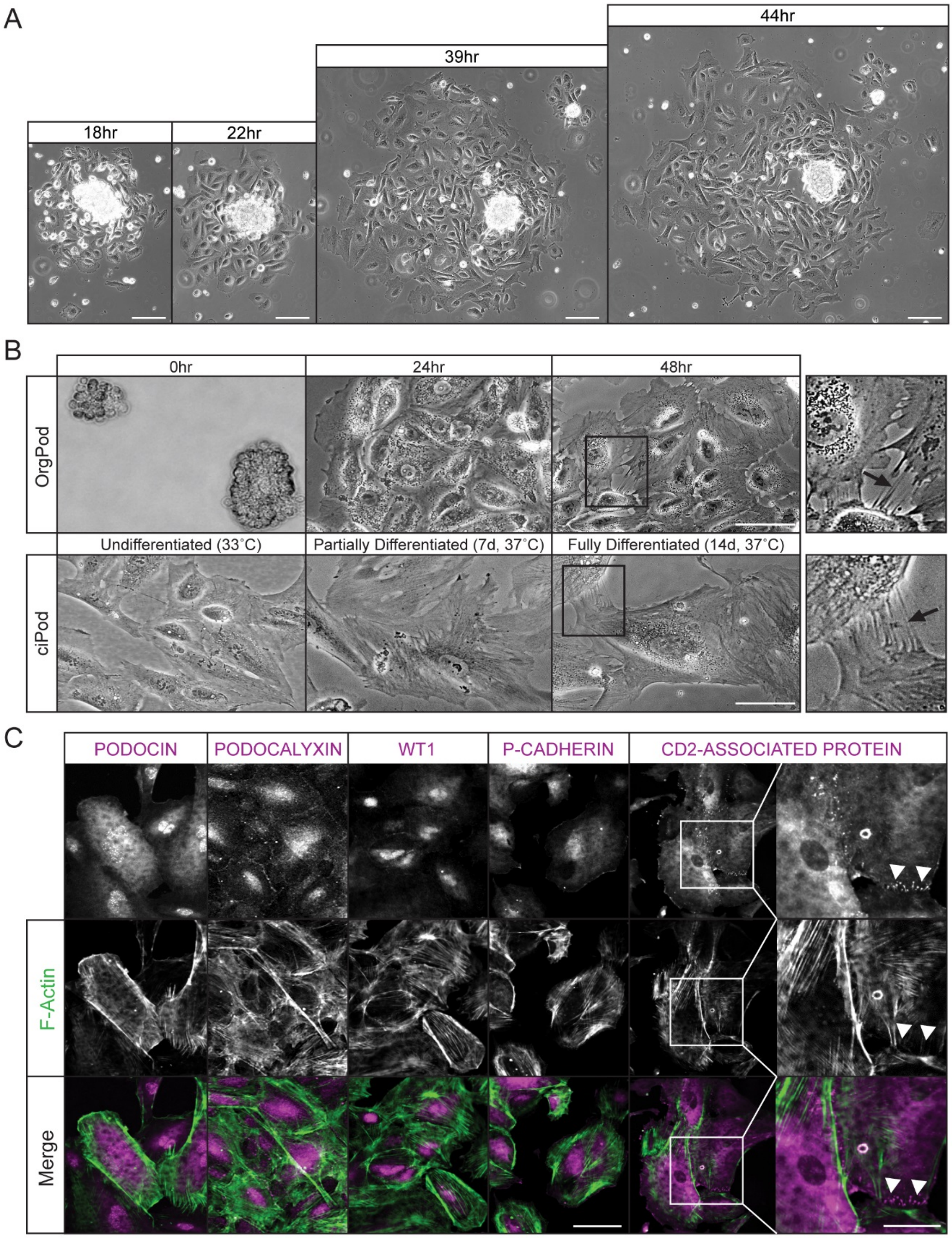

Supplementary Figure 1. Morphology and identity of primary podocytes isolated from organoid glomeruli. **A.** Organoid-derived podocytes (OrgPods) actively migrate from organoid glomeruli (OrgGloms) over time, forming a progressively more arborized monolayer.

Scale bars 100µm. **B.** Organoid-derived podocytes (OrgPods) in culture show comparable morphology to the gold-standard conditionally immortalised human podocyte cell line (ciPod) [2], displaying a flat arborized morphology with processes connecting adjacent cells. Scale bars 50µm. **C.** Fluorescent visualisation of classical podocyte markers alongside phalloidin to mark F-actin showed appropriate expression and localisation of these proteins within the OrgPod cell population (arrowheads mark areas of interest). PODOCIN showed a cytoplasmic distribution with increased levels at cell edges; PODOCALYXIN showed a punctuated distribution across the cell with focussed expression at cell junctions. WILMS' TUMOUR was predominately nuclear, as anticipated, whilst P-CADHERIN, another marker of differentiated cells, was cytoplasmic, perinuclear and also present at the cell surface. SYNAPTOPODIN was found in close association with stress fibres of the actin cytoskeleton whilst CD2-ASSOCIATED PROTEIN showed mature expression in areas of cell-cell contact at the edges of F-actin tips. Representative immunofluorescence images shown in greyscale for single channels, merged images in colour. Scale bar 50µm.

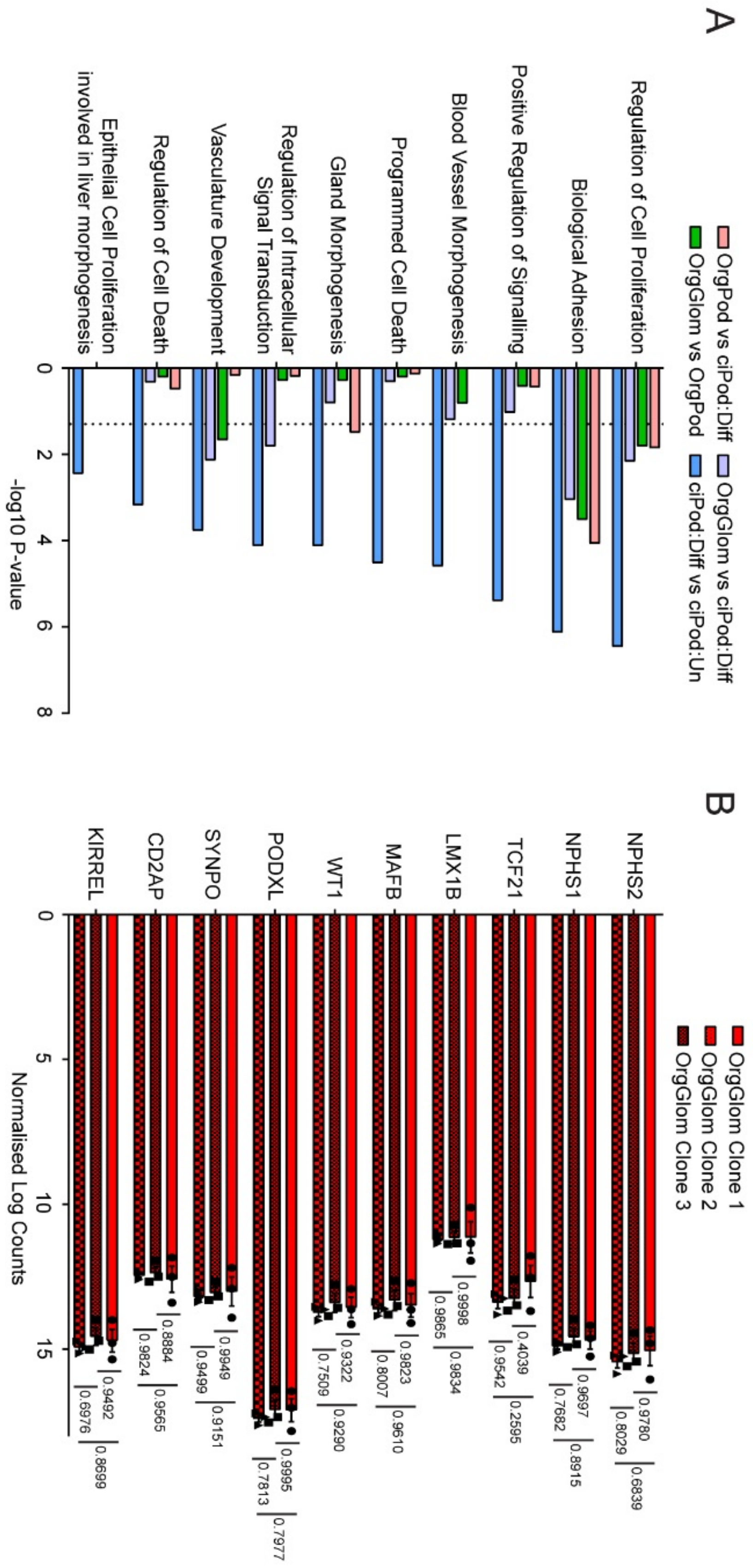

Supplementary Figure 2. Transcriptional comparison of organoid glomeruli with podocytes cultured in 2D. **A.** Gene Ontology (GO) enrichment analysis of the top 100 differentially

expressed genes upregulated between differentiated and undifferentiated ciPods. The top 10 significant GO terms based on *P* value are presented. *P* value of 0.05 shown as dotted line. **B.** The reproducibility of the OrgGlom model using clones derived from 3 different iPSC lines was highlighted by podocyte gene expression levels in a targeted gene panel. Clone 1 represents the line used for all other subsequent analyses. Two way ANOVA, between clones p=0.1943; error bars = S.E.M.; significant difference between clones assessed by Tukey's multiple comparisons test; F-value=1.684; DF=2; biological replicates n=3.

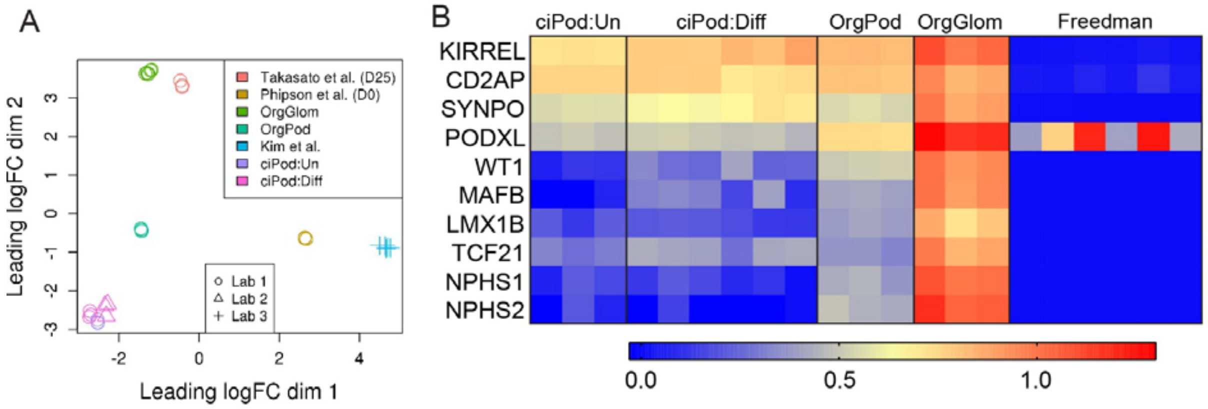

Supplementary Figure 3. Transcriptional comparison of organoids, OrgGloms, ciPods and undifferentiated iPSC. **A.** Principle component analysis of RNA sequencing (RNA-Seq) data was performed on data from Day 7+18 (Day 25) total kidney organoids [3], undifferentiated iPSC lines [4], OrgGloms, OrgPods and ciPods in both differentiated and undifferentiated states (our laboratory and a previously published ciPod data (Lab 2, [5]), in addition to data collected from Kim et al (Lab 3, [6]). All biological replicates are indicated as individual points. **B.** Heatmap illustrating relative expression of key podocyte genes in OrgGloms and undifferentiated iPSC [6].

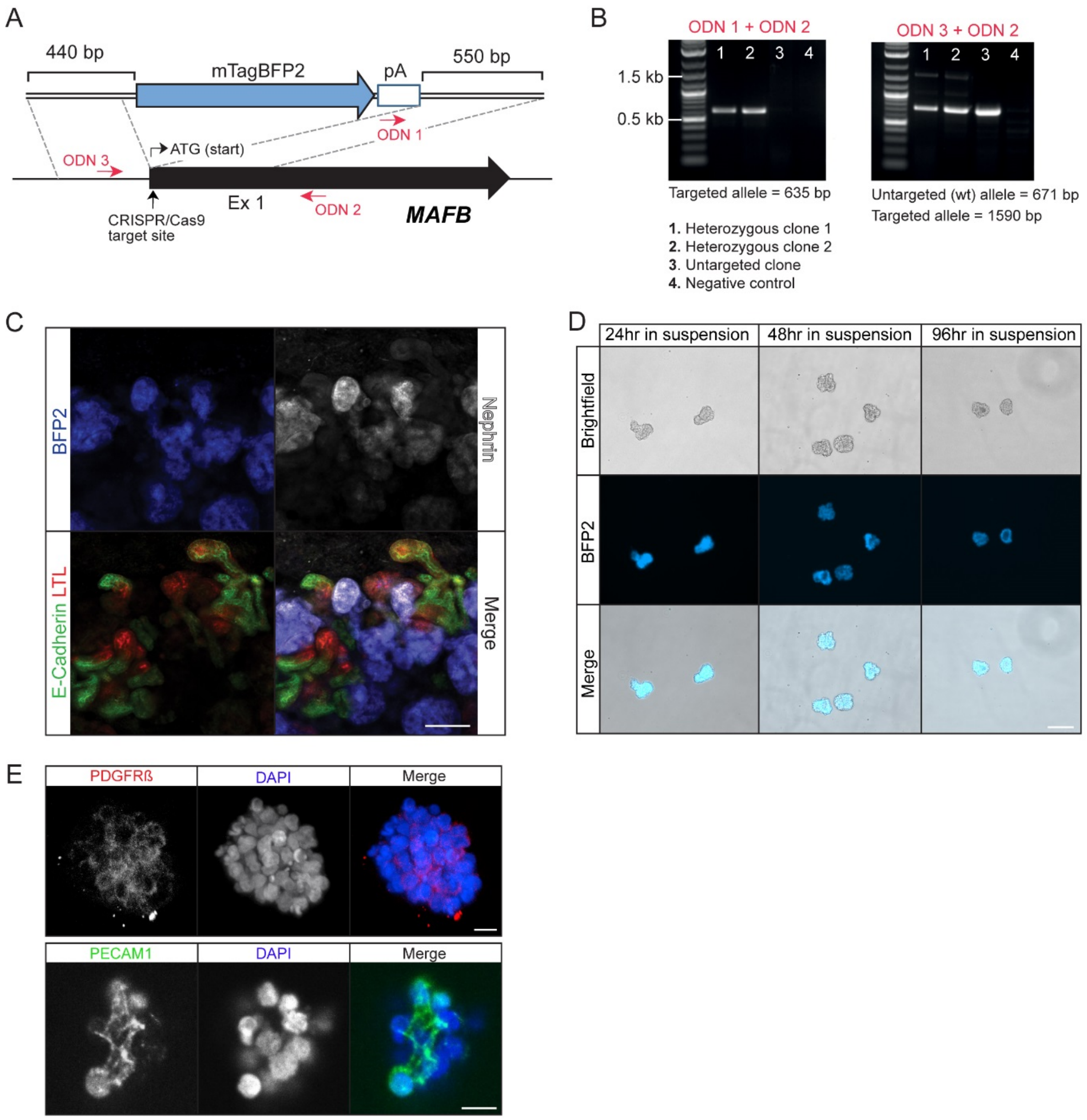

Supplementary Figure 4. Use of a human MAFB reporter iPSC line to analyse organoid-derived podocytes and glomeruli. **A.** Design of human MAFB-BFP2 reporter iPSC lines. Schematic diagram of the MAFB locus and the homologous template used for gene targeting. The oligonucleotides (ODNs) used for identifying targeted clones by PCR are indicated. pA, polyA signal; Ex, exon. **B.** Gel analysis of the PCR products amplified from correctly targeted MAFB clones. Primers ODN-1 and ODN-2 flank the recombination site with ODN-3 binding within the reporter cassette and ODN-2 binding outside the region of homology. **C.** MAFB-BFP2 iPSC reporter cell line was successfully directly differentiated into kidney organoid. Immunostaining of fixed organoids showed appropriate nephron segmentation with lotus tetragonolobus lectin (LTL) marking proximal tubule and E-CADHERIN marking maturing

proximal tubule, distal tubule and collecting duct. MAFB-BFP2 signal was found to overlay with NEPHRIN in the glomeruli confirming the specificity of the MAFB-tagged line. Scale bar 200µm. **D.** Live imaging of MAFB-BFP2 reporter expression over time in sieved OrgGloms shows robust gene expression when in suspension up to 96 hours post-isolation. **E.** Immunostaining of isolated OrgGloms shows low level expression of the endothelial marker PLATELET ENDOTHELIAL CELL ADHESION MOLECULE (*PECAM1*) and mesangial marker PLATELET-DERIVED GROWTH FACTOR RECEPTOR BETA (*PDGFRβ*). Expression levels of single channels shown in greyscale to preserve maximum contrast, merged images shown in colour. Scale bars 10µm.

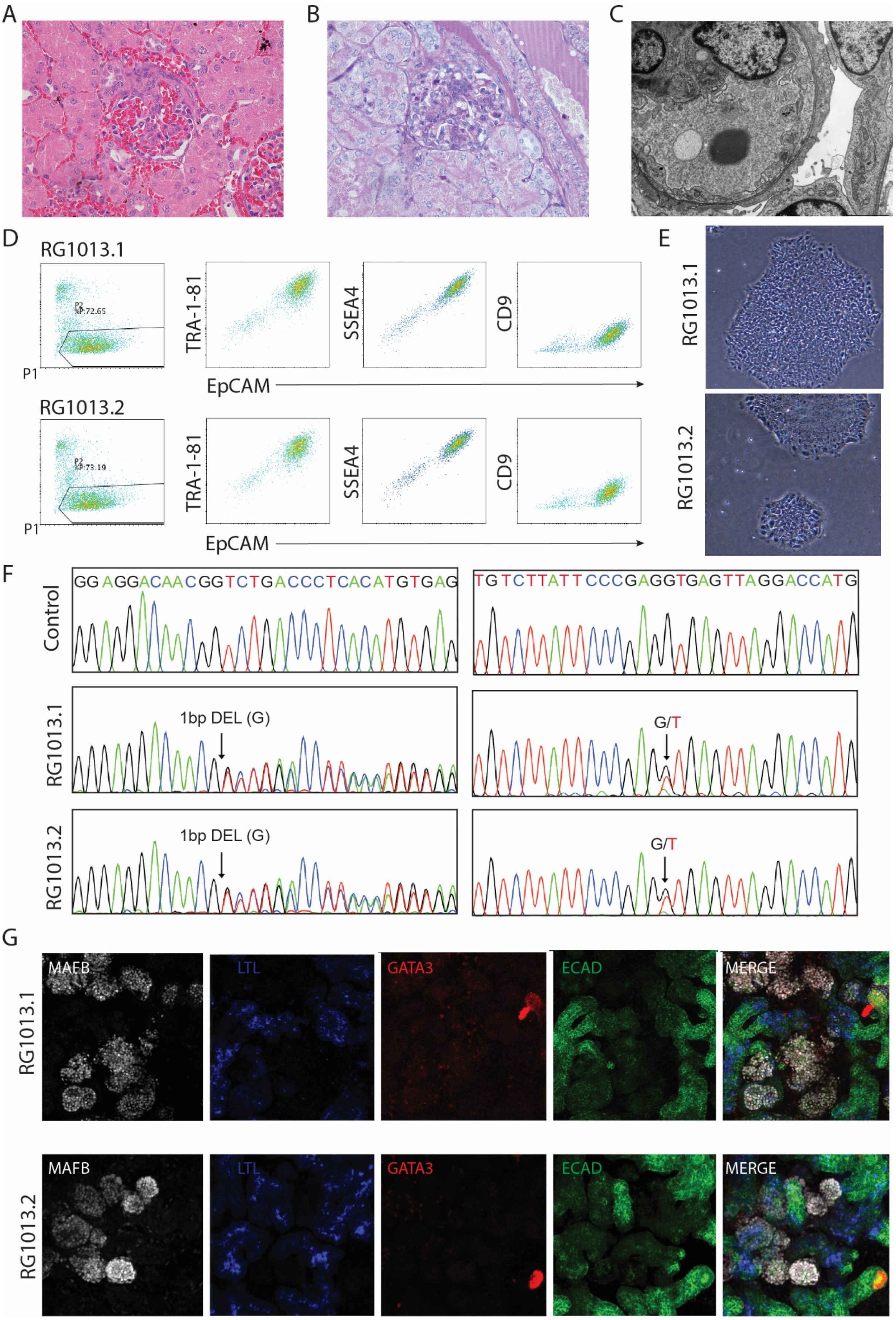
A
B
C
D
RG1013.1
P2
P1
TRA-1-81
SSEA4
CD9
EpCAM
RG1013.2
P2
P1
TRA-1-81
SSEA4
CD9
EpCAM
E
RG1013.1
RG1013.2
F
Control
GG AGGACAACGGTCTGACCCTCACATGTGAG
TGTCTTATTCCCGAGGTGAGTTAGGACCATG
RG1013.1
1bp DEL (G)
G/T
RG1013.2
1bp DEL (G)
G/T
G
RG1013.1
MAFB
LTL
GATA3
ECAD
MERGE
RG1013.2
MAFB
LTL
GATA3
ECAD
MERGE

Supplementary Figure 5. Clinical characterisation of congenital nephrotic syndrome patient and derivation and validation of patient iPSC clones. **A, B.** Haematoxylin and eosin (**A**) and PAS (**B**) stained sections of kidney tissue from congential nephrotic syndrome patient. **C.** TEM of podocyes from congenital nephrotic syndrome patient RG1013 kidney tissue revealing evidence for podocyte effacement and loos of microvilli. **D.** FACS analysis of pluripotency cell surface markers for patient-derived iPSC clones RG1013.1 and RG1013.2. SNP analysis was performed to validate the chromosomal integrity of both clones. No aneuploidies were detected. **E.** Brightfield images of patient-derived iPSC clones RG1013.1 and RG1013.2 in culture showing appropriate morphology for a pluripotent stem cell population. **F.** DNA sequencing was performed on both RG1013.1 and 1013.2 to validate the presence of the original patient mutations in exons 10 and 27. Chromatograms are presented for both regions showing clear evidence of a heterozygous mutation in these iPSC clones. **G.** Generation of appropriately patterned kidney organoids from both RG1013.1 and RG1013.2 iPSC. Immunofluorescence staining revealed the presence of appropriately patterned nephrons as evidenced by the presence of podocytes (MAFB, white), proximal segments (LTL, blue), distal segments (CDH1, green) and collecting duct (GATA3/CDH1, red/green).

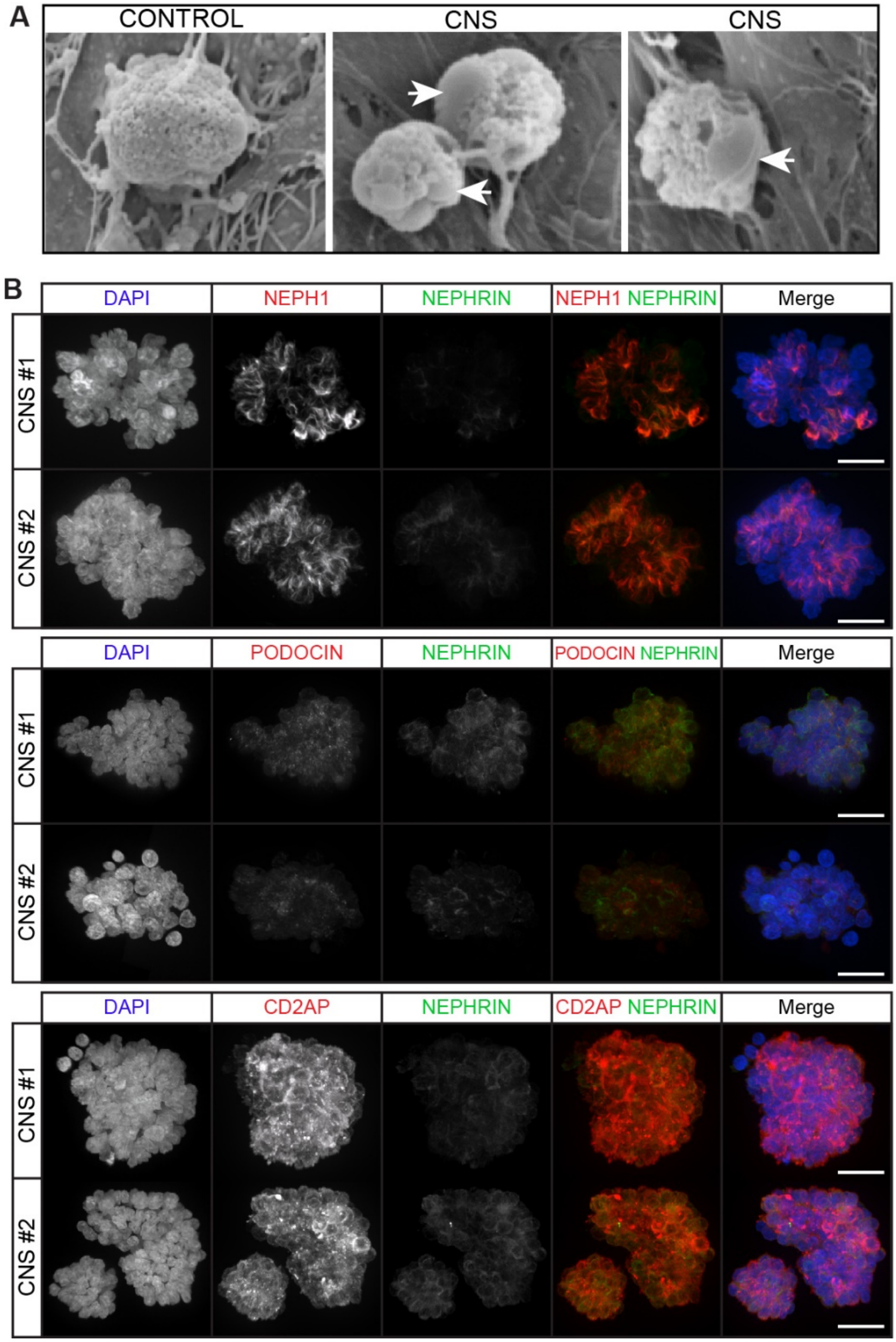

Supplementary Figure 6. Characterisation of organoid glomeruli from congenital nephrotic syndrome patient lines. **A.** Scanning electron microscopy of glomeruli within organoids derived from a control iPSC line (MAFBmTAGBFP reporter line) and a congenital nephrotic

syndrome (CNS) patient-derived iPSC line. Arrows indicate broad, flattened podocytes within patient glomeruli. Magnification 8000x. **B.** Immunostaining of OrgGloms isolated from CNS patient organoids derived from both iPSC clones (CNS#1: RG1013.1, CNS#2: RG1013.2) show comparable protein intensity, representative images shown. Scale bars 10µm.

Supplementary Video 1. Three-dimensional video of immunostained whole sieved organoid glomerulus. Confocal z-stack images were reconstructed to form a 3D image of the organoid glomerulus, immunostained for the podocyte proteins NEPHRIN (green), NEPH1 (red) and Podocalyxin (magenta) in addition to nuclei marked with DAPI (blue). Co-localisation of NEPHRIN and NEPH1 can clearly be observed at the basal junction between cells.

Supplementary references:

1. Lindenmeyer, M.T., et al., *Systematic analysis of a novel human renal glomerulus-enriched gene expression dataset.* PLoS One, 2010. **5**(7): p. e11545.
2. Saleem, M.A., et al., *A conditionally immortalized human podocyte cell line demonstrating nephrin and podocin expression.* J Am Soc Nephrol, 2002. **13**(3): p. 630-8.
3. Takasato, M., et al., *Generation of kidney organoids from human pluripotent stem cells.* Nat Protoc, 2016. **11**(9): p. 1681-92.
4. Phipson, B., et al., *Transcriptional evaluation of the developmental accuracy, reproducibility and robustness of kidney organoids derived from human pluripotent stem cells.* Nature Methods, 2018. **In Press**.
5. Jiang, L., et al., *RNA sequencing analysis of human podocytes reveals glucocorticoid regulated gene networks targeting non-immune pathways.* Sci Rep, 2016. **6**: p. 35671.
6. Kim, Y.K., et al., *Gene-Edited Human Kidney Organoids Reveal Mechanisms of Disease in Podocyte Development.* Stem Cells, 2017.
