# Supplementary material for: 3D organoid-derived human glomeruli for personalised podocyte disease modelling and drug screening
Source: Nat Commun. 2018 Dec 4;9:5167. doi: 10.1038/s41467-018-07594-z (PMC6279764; doi:10.1038/s41467-018-07594-z)
Supplement: Supplementary file 3 — Description of Additional Supplementary Files [file 41467_2018_7594_MOESM3_ESM.pdf]

Description of additional supplementary information

Supplementary Movie 1. Three-dimensional video of immunostained whole sieved organoid glomerulus. Confocal z-stack images were reconstructed to form a 3D image of the organoid glomerulus, immunostained for the podocyte proteins NEPHRIN (green), NEPH1 (red) and Podocalyxin (magenta) in addition to nuclei marked with DAPI (blue). Co-localisation of NEPHRIN and NEPH1 can clearly be observed at the basal junction between cells.
