# Supplementary material for: 3D organoid-derived human glomeruli for personalised podocyte disease modelling and drug screening
Source: Nat Commun. 2018 Dec 4;9:5167. doi: 10.1038/s41467-018-07594-z (PMC6279764; doi:10.1038/s41467-018-07594-z)
Supplement: Supplementary file 5 — Reporting Summary [file 41467_2018_7594_MOESM5_ESM.pdf]

nature research

Corresponding author(s): Melissa H Little

Reporting Summary

Nature Research wishes to improve the reproducibility of the work that we publish. This form provides structure for consistency and transparency in reporting. For further information on Nature Research policies, see Authors & Referees and the Editorial Policy Checklist.

Statistical parameters

When statistical analyses are reported, confirm that the following items are present in the relevant location (e.g. figure legend, table legend, main text, or Methods section).

n/a	Confirmed	
☐	☒	The exact sample size (n) for each experimental group/condition, given as a discrete number and unit of measurement
☐	☒	An indication of whether measurements were taken from distinct samples or whether the same sample was measured repeatedly
☐	☒	The statistical test(s) used AND whether they are one- or two-sided<br>*Only common tests should be described solely by name; describe more complex techniques in the Methods section.*
☒	☐	A description of all covariates tested
☐	☒	A description of any assumptions or corrections, such as tests of normality and adjustment for multiple comparisons
☐	☒	A full description of the statistics including central tendency (e.g. means) or other basic estimates (e.g. regression coefficient) AND variation (e.g. standard deviation) or associated estimates of uncertainty (e.g. confidence intervals)
☐	☒	For null hypothesis testing, the test statistic (e.g. F, t, r) with confidence intervals, effect sizes, degrees of freedom and P value noted<br>*Give P values as exact values whenever suitable.*
☒	☐	For Bayesian analysis, information on the choice of priors and Markov chain Monte Carlo settings
☐	☐	For hierarchical and complex designs, identification of the appropriate level for tests and full reporting of outcomes
☐	☐	Estimates of effect sizes (e.g. Cohen's d, Pearson's r), indicating how they were calculated
☐	☒	Clearly defined error bars<br>*State explicitly what error bars represent (e.g. SD, SE, CI)*

Our web collection on statistics for biologists may be useful.

Software and code

Policy information about availability of computer code

Data collection	Transcript levels quantification of Illumina NextSeq 500 RNA-seq data was obtained by pseudoalignment to the GRCh38 genome using Salmon (v.0.8.2), and converted to gene counts with tximport v(1.4.0). Quantitative PCR was performed with SensiFAST SYBR No-ROX cDNA synthesis kit (Bioline) by the 7500 Real Time PCR System Thermal Cycler (Applied Biosystems) machine.
Data analysis	R (v 3.5) and PRSIM (v 7) was used for analysis and visualization of the bulk RNA-seq expression and PCR data. R packages from Bioconductor and CRAN used for specific RNA-seq analysis were: AnnotationDbi (v1.40.0), Biobase (v2.36.2), biomaRt (v2.37.6) , edgeR (v3.18.1), ggplot2(v2.2.1), limma (v3.34.9), NMF (v0.21.0), RColorBrewer (v1.1-2), org.Hs.eg.db (v3.4.1), gcrma (v2.5.0), hgu133a2.db (3.2.3), and annotate (v1.54.0).

For manuscripts utilizing custom algorithms or software that are central to the research but not yet described in published literature, software must be made available to editors/reviewers upon request. We strongly encourage code deposition in a community repository (e.g. GitHub). See the Nature Research guidelines for submitting code & software for further information.

Data

Policy information about availability of data

All manuscripts must include a data availability statement. This statement should provide the following information, where applicable:

- Accession codes, unique identifiers, or web links for publicly available datasets
- A list of figures that have associated raw data
- A description of any restrictions on data availability

The authors can confirm that all relevant data are included in the paper and/or its supplementary information files. RNAseq data generated within this study have been deposited in the NCBI Gene Expression Omnibus and will be publicly accessible using the GEO accession codes listed in the manuscript. All proteomic data has been submitted to the PRIDE PRoteomics IDEntification database (https://www.ebi.ac.uk/pride) and will be publicly accessibly using the provided accession code. All other original data pertaining to this manuscript are available from within the Source Data file or from the authors directly.

Field-specific reporting

Please select the best fit for your research. If you are not sure, read the appropriate sections before making your selection.

☒ Life sciences ☐ Behavioural & social sciences ☐ Ecological, evolutionary & environmental sciences

For a reference copy of the document with all sections, see nature.com/authors/policies/ReportingSummary-flat.pdf

Life sciences study design

All studies must disclose on these points even when the disclosure is negative.

Sample size	All samples evaluated at the level of RNAseq were performed in triplicate to facilitate a statistical evaluation of differential expression. Data presented in Figure 6 represents the analysis of a minimum of 8, a maximum of 56 OrgGloms isolated from each of three distinct iPSC differentiations, including two independant iPSC clones generated from the same patient. Data presented in Figure 7 represents a total of 16 measurements of individual OrgGloms at each concentration of doxorubicin.
Data exclusions	No data were excluded
Replication	Organoids were generated from a total of 6 distinct iPSC lines within this manuscript, the origin of which has been described in all instances. DNA sequencing is presented to validate the presence of both patient-specific mutations in both clones from the described congenital nephrotic syndrome patient. All iPSC lines, including patient and reporter clones, were validated for pluripotency, SNP validated to ensure the absence of any genomic rearrangements and tested for their capacity to differentiate into kidney organoids.
Randomization	This does not apply for this study.
Blinding	It was not always possible to be blinded as not all differentiations were performed at the same time or analysed in the same way. In the case of evaluating control versus patient-derived clone 1013.1, the fluorescence intensity quantification was performed blinded. The second clone was a subsequent differentiation and was measured in isolation also verses a control. In the case of the SEM performed on the control and patient line 1013.1, the operator was blinded to the source of the glomeruli.

Reporting for specific materials, systems and methods

Materials & experimental systems

n/a	Involved in the study	
☐	☒	Unique biological materials
☐	☒	Antibodies
☒	☐	Eukaryotic cell lines
☒	☐	Palaeontology
☒	☐	Animals and other organisms
☐	☒	Human research participants

Methods

n/a	Involved in the study	
☒	☐	ChIP-seq
☐	☒	Flow cytometry
☒	☐	MRI-based neuroimaging

Unique biological materials

Policy information about availability of materials

Obtaining unique materials	This study involved the use of patient-derived iPSC lines. Ethics approval for the derivation and characterisation of these lines limits the distribution of this material. Research using these lines is restricted to the analysis of kidney disease performed by the researchers listed on the human ethics approval.

Antibodies

Antibodies used	All antibodies used in this study are commercially available.
Validation	All antibodies have been previously used on human material and are noted as suitable for this purpose by the distributor. We have previously published the application of these antibodies in Takasato et al, 2015, Nature.

Human research participants

Policy information about studies involving human research participants

Population characteristics	Only a single patient was involved in this study. Two iPSC clones were derived from peripheral blood mononuclear cells using Sendai virus reprogramming. This material was collected with consent and under the human ethics approval number HREC/15/QRCH/126.
Recruitment	The patient presented at Royal Childrens Hospital and was recruited into the KidGen consortium for diagnostic genomics. After identification of a known and a likely disease causing variant in NPHS1, the patient was consented for iPSC derivation.

Flow Cytometry

Plots

Confirm that:

☒ The axis labels state the marker and fluorochrome used (e.g. CD4-FITC).

☒ The axis scales are clearly visible. Include numbers along axes only for bottom left plot of group (a 'group' is an analysis of identical markers).

☒ All plots are contour plots with outliers or pseudocolor plots.

☒ A numerical value for number of cells or percentage (with statistics) is provided.

Methodology

Sample preparation	iPSC-derived kidney organoids were dissociated by incubation with TrypLE select enzyme (Thermo Fisher Scientific) for 10 minutes at 37°C, with gentle pipetting every 2 minutes to aid dissociation. Cells were then passed through a series of cell strainers with sequentially smaller mesh sizes, ranging from 100µM to 40µM (pluriSelect), in order to obtain a single cell population. mTagBFP2 positive OrgGloms were captured and isolated from within the sieves. The remaining single cell population was then sorted for mTagBFP2 signal.
Instrument	FACSAria Fusion flow cytometer (BD Biosciences).
Software	FACsDIVA (BD Biosciences) and Flowlogic (Inivia)
Cell population abundance	d7+10 samples, 3 biological replicates: BFP2+ populations = 1.69x10e6, 1.80x10e6 and 2.0x10e6 respectively.<br>d7+14 samples, 3 biological replicates: BFP2+ populations = 0.56x10e6, 0.49x10e6 and 0.45x10e6 respectively.<br>d7+19 samples did not have sufficient BFP2+ cell populations to collect due to very low single cell abundance.
Gating strategy	See Data Source file.

☒ Tick this box to confirm that a figure exemplifying the gating strategy is provided in the Supplementary Information.
